# Distinctive Structure and Assembly of Phyllosphere Microbial Communities between Wild and Cultivated Rice

**DOI:** 10.1128/spectrum.04371-22

**Published:** 2023-01-10

**Authors:** Yue Yin, Yi-Fei Wang, Hui-Ling Cui, Rui Zhou, Lv Li, Gui-Lan Duan, Yong-Guan Zhu

**Affiliations:** a State Key Lab of Urban and Regional Ecology, Research Center for Eco-Environmental Sciences, Chinese Academy of Sciences, Beijing, China; b University of Chinese Academy of Sciences, Beijing, China; c Institute of Urban Environment, Chinese Academy of Sciences, Xiamen, China; University of Massachusetts Amherst

**Keywords:** wild rice, phyllosphere, microbial community, core microbiome, community assembly, cooccurrence network, *Methylobacterium*

## Abstract

Wild rice has been demonstrated to possess enriched genetic diversity and multiple valuable traits involved in disease/pest resistance and abiotic stress tolerance, which provides a potential resource for sustainable agriculture. However, unlike the plant compartments such as rhizosphere, the structure and assembly of phyllosphere microbial communities of wild rice remain largely unexplored. Through amplicon sequencing, this study compared the phyllosphere bacterial and fungal communities of wild rice and its neighboring cultivated rice. The core phyllosphere microbial taxa of both wild and cultivated rice are dominated with *Pantoea*, *Methylobacterium*, *Nigrospora*, and *Papiliotrema*, which are potentially beneficial to rice growth and health. Compared to the cultivated rice, *Methylobacterium*, *Sphingomonas*, *Phaeosphaeria*, and *Khuskia* were significantly enriched in the wild rice phyllosphere. The potentially nitrogen-fixing *Methylobacterium* is the dominated wild-enriched microbe; *Sphingomonas* is the hub taxon of wild rice networks. In addition, the microbiota of wild rice was more governed by deterministic assembly with a more complicated and stable community network than the cultivated rice. Our study provides a list of the beneficial microbes in the wild rice phyllosphere and reveals the microbial divergence between wild rice and cultivated rice in the original habitats, which highlights the potential selective role of wild rice in recruiting specific microbiomes for enhancing crop performance and promoting sustainable food production.

**IMPORTANCE** Plant microbiota are being considered a lever to increase the sustainability of food production under a changing climate. In particular, the microbiomes associated with ancestors of modern cultivars have the potential to support their domesticated cultivars. However, few efforts have been devoted to studying the biodiversity and functions of microbial communities in the native habitats of ancestors of modern crop species. This study provides a list of the beneficial microbes in the wild rice phyllosphere and explores the microbial interaction patterns and the functional profiles of wild rice. This information could be useful for the future utilization of the plant microbiome to enhance crop performance and sustainability, especially in the framework of sustainable agroecosystems.

## INTRODUCTION

There are diverse microorganisms colonizing on plant leaves, including bacteria, fungi, archaea, protists, and viruses (among which bacteria and fungi are dominant) (1 to 3). It has been widely reported that the phyllosphere microbiome can not only enhance the development and health of host plants, but also play crucial roles in the Earth’s biogeochemical cycles and attenuate the residues of pesticides and atmospheric pollutants (4 to 6). The assembly of the phyllosphere microbiome is influenced by both intrinsic plant factors (e.g., plant species and genotypes) and environmental conditions (e.g., temperature, solar radiation, humidity, soil type, and agricultural activity), while biotic and abiotic selection pressures must also be considered (4). Among these plant-associated microbiota, the core microbiomes, which are consistently associated with a particular crop species under all environmental conditions, are emerging as attractive. The core microbiome has been considered to have coevolved with host plants, and its persistent occurrence and related functions could ensure plant health and productivity (7, 8). Therefore, a more comprehensive knowledge about the assembly and composition of the phyllosphere core microbiome of crops would be beneficial for promoting crop management and productivity.

Rice (Oryza sativa *L.*) is one of the most important cereal crops, feeding more than half of the world’s population (9). With the increase of the world’s population, the current growth of agricultural production does not meet what is needed to maintain global food security (10, 11). Moreover, intense agronomic practices (such as pesticides and chemical fertilizers) have led to soil degradation and environment deterioration, which further threaten the food supply (12, 13). Clearly, there is an urgent need to develop ecological intensification strategies for sustainable rice production. For this purpose, rice-associated microbiomes have been attracting attentive research. It has been reported that the microbiomes associated with ancestors of modern cultivars have the potential to support their domesticated cultivars, which is considered an alternative to agrochemical use without environmental costs (14, 15). Common wild rice (*Oryza rufipogon Griff.*), the wild progenitor of cultivated rice, has been reported to have various unique traits, such as disease resistance and lodging and drought tolerance (16 to 18). However, recent studies revealed that during the process of domestication, crop plants may not only lose genetic diversity, but also lose some host-specific microbes, which may be recruited by the distinct traits of wild relatives (14, 19). So far, the rhizosphere microbial communities of wild rice have been preliminarily characterized (20), while the phyllosphere microbiome of wild rice has rarely been documented. Therefore, it is crucial to gain a better understanding of the phyllosphere microbial community of wild rice, which is also essential to modulate its activity for the benefit of rice production.

Understanding the ecological patterns and community features will provide insights into the formation of plant-microbe holobionts and management of microbial communities for enhanced ecosystem service provisioning (21). Ecological theories suggest that the formation of microbial communities is governed by complex interactions among microbes, host, and environment (22). Microorganisms, which have microscopic sizes and high dispersal capacity, could also exhibit complex relations within an ecological niche (23). Network-based cooccurrence pattern analysis has recently been used in diverse habitats and helped to disentangle these complex interactions (24, 25). In addition, from the metacommunity perspective, microbial community assembly is a comprehensive result of deterministic and stochastic processes, including selection, dispersal limitation, homogenizing dispersal, and drift (26, 27). Furthermore, at larger horizontal scales, selection and dispersal limitation are the main processes governing microbial community assembly, while homogenizing dispersal and drift are reputed to play a minor role (27, 28). So far, the patterns of species coexistence, and the relative importance of different assembly processes in establishing and maintaining wild rice phyllosphere microbial communities remains unknown.

In the present study, the leaves of wild rice and its neighboring cultivated rice were sampled from different sites in China, and the phyllosphere microbial communities were characterized through high-throughput sequencing. The aims of our study were to (i) identify the core microbiome in the phyllosphere of wild and cultivated rice; (ii) compare the rice phyllosphere microbial community between wild progenitors versus modern cultivars; and (iii) reveal the cooccurrence patterns and community assembly processes of phyllosphere microbial communities in wild and cultivated rice. We hypothesize that there are distinctive structure and assembly of the phyllosphere microbial communities between wild and cultivated rice.

## RESULTS

### Characterization of microbial communities of the rice phyllosphere.

**FIG 1:**
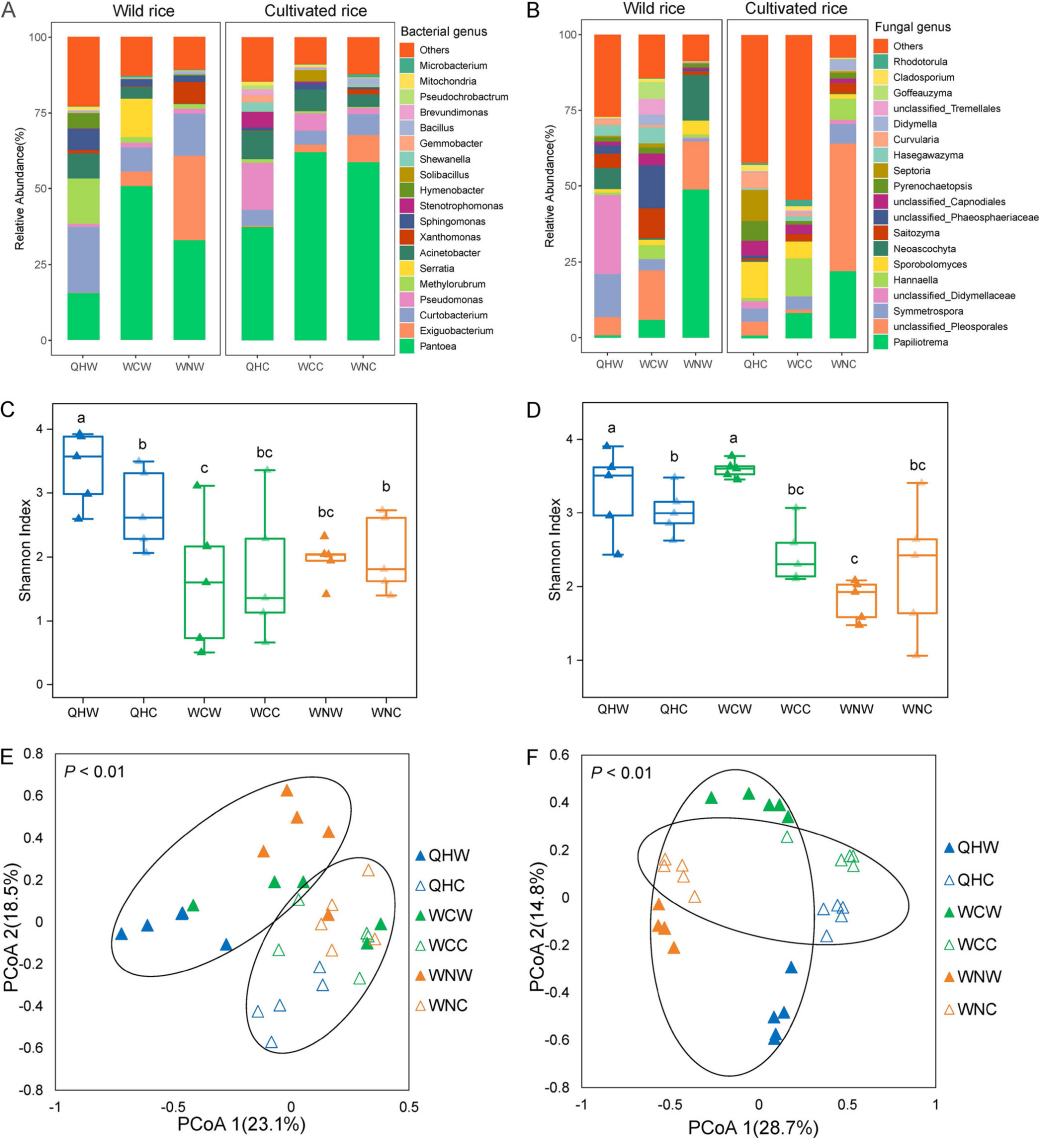
Taxonomic profiles of microbial communities in wild and cultivated rice phyllosphere from different sites. The relative abundance of bacteria (A) and fungi (B) at genus level present in the rice phyllosphere. The Shannon index of bacterial (C) and fungal (D) communities in each rice type. The PCoA analysis of bacterial (E) and fungal (F) communities based on Bray-Curtis distances grouped by rice types and sites. (QHW, wild rice in Qionghai; WCW, wild rice in Wenchang; WNW, wild rice in Wanning; QHC, cultivated rice in Qionghai; WCC, cultivated rice in Wenchang; WNC, cultivated rice in Wanning.)

A total of 2,714,616 bacterial and 3,281,731 fungal high-quality sequencing reads were obtained across all samples, which were clustered into 7,423 bacterial amplicon sequence variants (ASVs) and 3,874 fungal ASVs, respectively. The dominant bacterial genera were *Pantoea*, *Curtobacterium*, and *Exiguobacterium*, accounting for 42.9%, 10.0%, and 7.5% of the total sequences, respectively (Fig. 1A). The most abundant fungal genera were *Papiliotrema*, unclassified *Pleosporales*, and *Symmetrospora*, accounting for 14.5%, 14.4%, and 5.7%, respectively (Fig. 1B). The results of α-diversity analysis showed that the bacteria (Fig. 1C) and fungi (Fig. 1D) from the wild rice were more diverse than those from the cultivated rice sampled at the same site, except the fungi from the Wanning site (Fig. 1D). Principal coordinate analysis (PCoA) based on Bray-Curtis distance demonstrated that the composition of bacterial and fungal communities clearly separated according to both sampling sites and rice genotypes (Fig. 1E and F). Permutational multivariate analyses of variance (PERMANOVA) based on Bray-Curtis distances revealed that the sampling site exerted a significant effect on the total microbiome (*R^2^* = 0.21 for bacteria and *R^2^* = 0.36 for fungi, *P = *0.001 for both), followed by rice cultivar (*R^2^* = 0.13 for bacteria and *R^2^*= 0.13 for fungi, *P = *0.001 for both) (Tables S1 and S2 in the supplemental material).

### Identification of core microbial taxa of rice phyllosphere.

**FIG 2:**
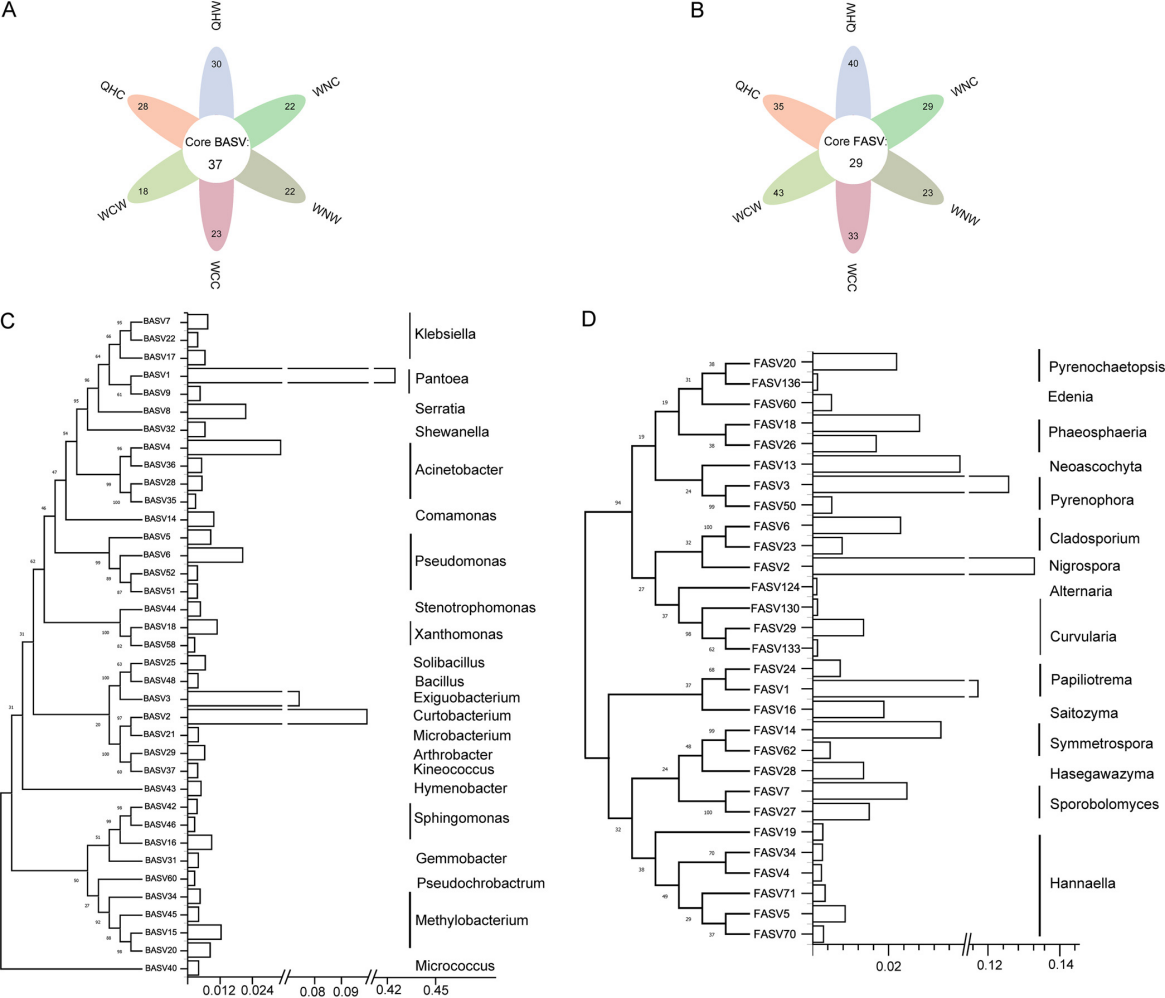
The shared core microbial taxa between wild and cultivated rice from different sites. Thirty-seven core bacterial taxa (A) and 29 core fungal taxa (B) were detected across all rice phyllosphere samples, as depicted by the Venn diagram. Neighbor-joining (NJ) phylogenetic trees and corresponding bar diagrams illustrating the 37 core bacterial (C) and 29 core fungal ASVs (D) (with 95% similarity as the cutoff value) and their relative abundances in all samples.

There were 37 bacterial and 29 fungal ASVs that were consistently present in all the samples (Fig. 2), which were considered the core microbial taxa. The core microbiome comprised only 0.5% and 0.8% of bacterial and fungal ASVs, while it accounted for 84.2% and 67.9% of relative abundance on average, respectively. The core rice phyllosphere bacteria contained 27 *Proteobacteria* ASVs that made up 62.8% of relative abundance, and *Pantoea* was the most abundant genus (2 ASVs, 42.7%), followed by *Methylobacterium* (4 ASVs, 4.8%), Pseudomonas (4 ASVs, 3.6%), Acinetobacter (4 ASVs, 2.9%), and *Serratia* (1 ASV, 2.1%) (Fig. 2C and Table S3). Except for *Proteobacteria*, *Actinobacteriota* (5 ASVs, 11.7% relative abundance), *Firmicutes* (3 ASVs, 8.5%), and *Bacteroidota* (1 ASV, 0.5%) were the abundant phyla of the core phyllosphere bacteria (Table S3). As for fungi, 29 core fungal ASVs were mainly classified in the genera of *Nigrospora* (13.2%), *Pyrenophora* (13.1%), *Papiliotrema* (12.5%), and *Phaeosphaeria* (4.5%) (Fig. 2D and Table S4). Although the core taxa were present in all the samples, the relative abundance of these ASVs varied significantly across rice species and sampling sites; for instance, the relative abundance of *Curtobacterium*, *Methylobacterium*, *Kineococcus*, and *Phaeosphaeria* in wild rice was significantly higher than that in cultivated rice (Fig. 2 and Fig. S2).

### The distinction of the phyllosphere microbiome between wild and cultivated rice.

**FIG 3:**
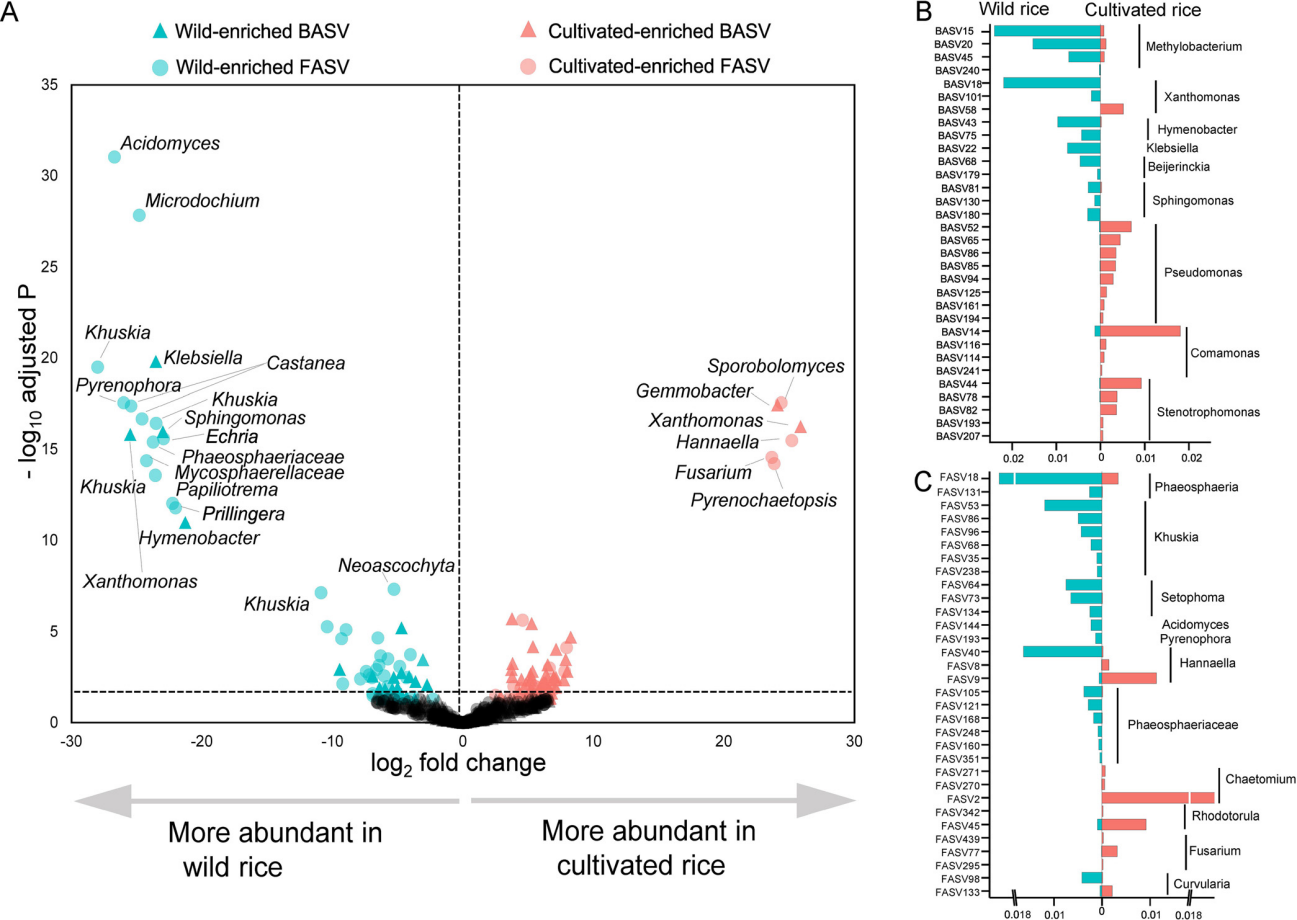
Specific differences of wild versus cultivated-enriched taxa. (A) Diverse bacterial and fungal taxa were enriched or depleted in the phyllosphere of wild rice relative to cultivated rice. Each point represents one amplicon sequence variant (ASV) and was labeled as the closest matching species based on comparison to the NCBI sequence database using BLAST. The relative abundance of main enriched phyllosphere BASVs (B) and FASVs (C) of wild rice and cultivated rice, respectively.

Through the differential abundance test between wild and cultivated rice, we identified a total of 71 significantly varied bacterial amplicon sequence variants (BASVs) (22 wild-enriched BASVs; 49 cultivated-enriched BASVs) and 65 significantly varied fungal amplicon sequence variants (FASVs) (41 wild-enriched FASVs; 24 cultivated-enriched FASVs) (|log_2_ fold change| >2, false discovery rate [FDR] <0.01) (Fig. 3 and Fig. S4). For bacteria, BASV18, BASV22, and BASV130 were mostly enriched in the wild rice, which were phylogenetically classified as *Xanthomonas*, Klebsiella and *Sphingomonas*, respectively (Fig. 3, Fig. S4A, and Table S5). Meanwhile, there were some core bacteria significantly enriched in the wild rice, such as *Methylobacterium* (BASV15, 20, 45), *Xanthomonas* (BASV18), *Hymenobacter* (BASV43), and Klebsiella (BASV22) (Fig. S3). Compared to the cultivated rice, *Methylobacterium* was the most enriched genus in wild rice (Fig. 3B). For fungi, FASV53, FASV144, and FASV193 were mostly enriched in the wild rice, which were phylogenetically classified as *Khuskia*, *Acidomyces*, and *Pyrenophora*, respectively, while none of the wild-enriched FASVs were core fungal taxa (Fig. 3, Fig. S4B and Table S6). Compared to the wild rice, Pseudomonas, *Comamonas*, *Chaetomium*, and *Rhodotorula* were the main enriched bacterial and fungal genera in the cultivated rice, respectively (Fig. 3, Tables S5 and S6).

### The predicted functional profiles of the rice phyllosphere microbiome.

Functional annotation of the prokaryotic taxa (FAPROTAX) indicated that the dominant functional groups of rice phyllosphere bacteria were associated with chemoheterotrophy and aerobic chemoheterotrophy (Fig. S5A), while the fungi were associated with the functional potentials of fungal parasites, plant pathogens, plant saprotrophy, and litter saprotrophy (Fig. S5B). Between wild and cultivated rice, significantly higher functional potentials of methanol oxidation (+594%), methylotrophy (+460%), ureolysis (+430%), and photoheterotrophy (+345%) were observed in wild rice than in cultivated rice (*P* < 0.05) (Fig. S5C). In addition, cultivated rice had significantly higher potentials of plant pathogens (+91%), human-associated (+88%) and human pathogens (+88%) microbial-associated processes (Fig. S5C). Among the phyllosphere fungi, a total of 160,247 high-quality sequences were identified to be potential fungal plant pathogens, which were affiliated with 58 genera, with relative abundances ranging from 3.0% to 7.3% (Fig. S6).

**FIG 4:**
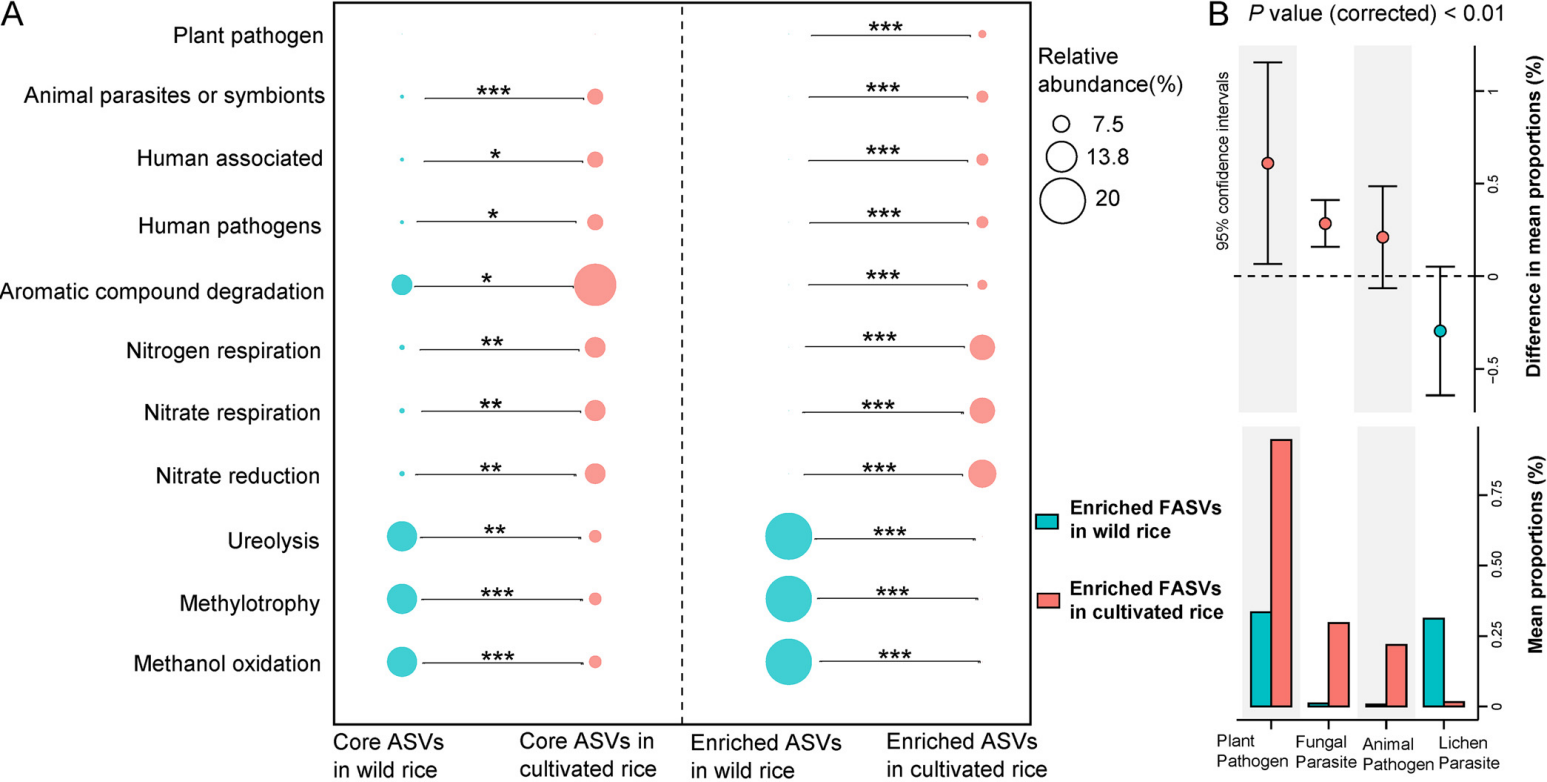
Predicted functional profiles of the rice phyllosphere microbiome. (A) Functional prediction of core and enriched BASVs in wild rice and cultivated rice using FAPROTAX, respectively. Only significant differences between wild and cultivated rice were compared using FDR-corrected Wilcoxon’s test (*, *P* < 0.05; **, *P* < 0.01). (B) Stamp analysis showed the significantly different fungal functional groups among enriched FASVs of wild rice and cultivated rice.

The core ASVs and the significantly varied ASVs are of special interest; thus, their functions were further analyzed (Fig. 4). Wild rice core BASVs showed significantly higher functional potentials of methylotrophy, methanol oxidation, and ureolysis, while lower functional potentials of human pathogens and animal parasites or symbionts, than those of cultivated rice, and wild-enriched BASVs showed the same trend (*P < *0.001) (Fig. 4A). For fungi, stamp analysis showed that plant pathogens, fungal parasites, and animal pathogens were significantly more abundant in cultivated-enriched FASVs than those in wild rice (*P < *0.01) (Fig. 4B).

### The network structure of rice phyllosphere microbiome.

Based on intrakingdom network analysis, the bacterial network of wild rice consisted of 249 nodes and 3,402 edges, whereas there were 240 nodes and 2,974 edges for cultivated rice (Fig. S7). The fungal network of wild rice consisted of 293 nodes and 4,727 edges, whereas there were 254 nodes and 2,734 edges for cultivated rice (Fig. S7). More nodes and edges, and higher average degree, in wild rice than cultivated rice suggest tighter associations among microbes in wild rice than in cultivated rice, and wild rice networks have more complex structures (Table S7). In addition, all the networks had overwhelmingly more positive associations than negative ones (Fig. S7).

**FIG 5:**
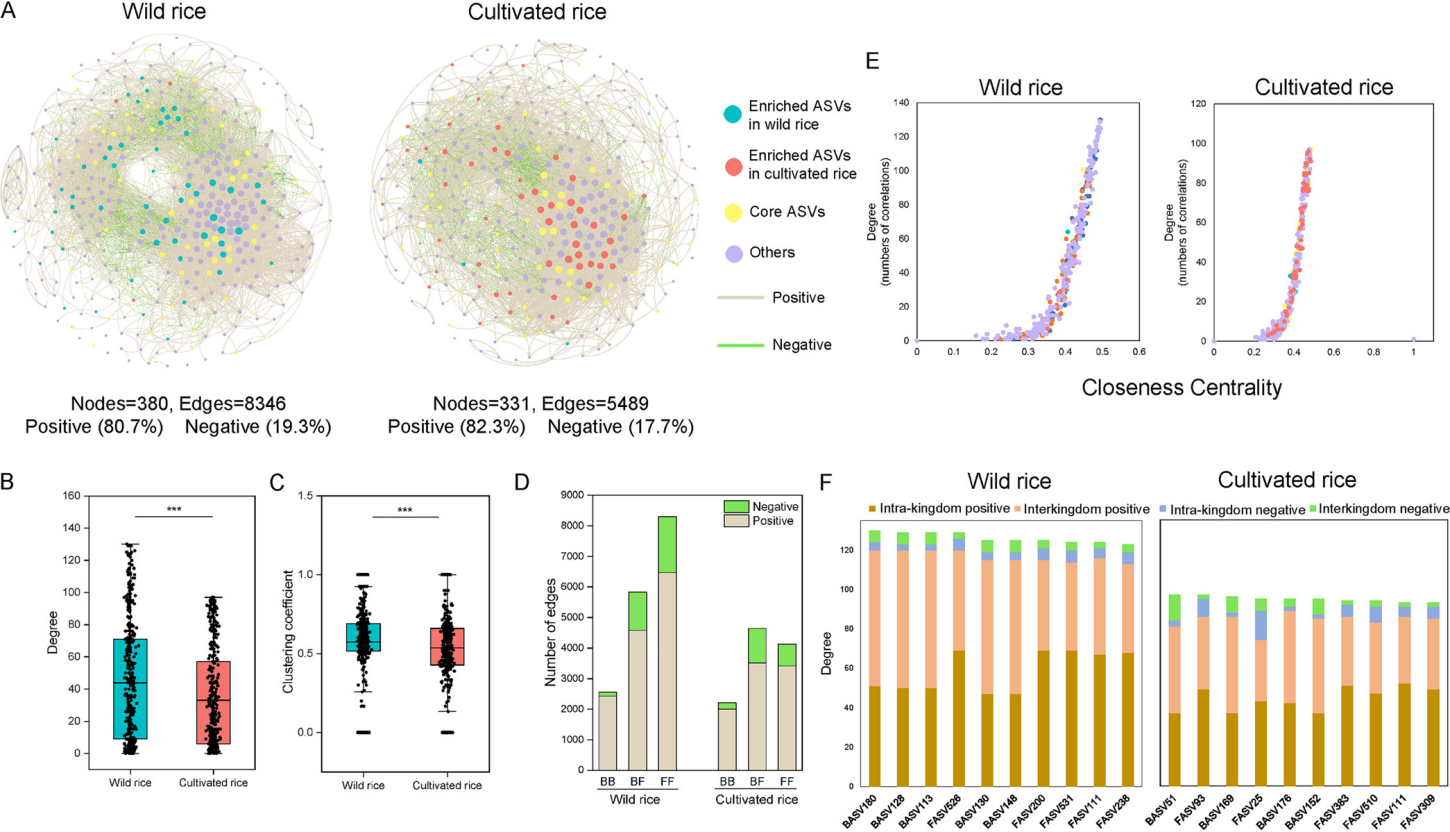
Interkingdom cooccurrence networks. (A) Networks contained both bacterial and fungal taxa showing a higher number of nodes and edges in the wild rice network than those in the cultivated rice network. Positive and negative relationships are illustrated in gray and green, respectively. Degree (B) and clustering coefficient values (C) of nodes and number of bacterial–bacterial (BB), bacterial–fungal (BF), and fungal–fungal (FF) correlations (D) in wild and cultivated rice networks. (E) Comparison of node-level topological features in panel A demonstrating the high degree and closeness centrality values for the hub taxa. Taxonomic information of the hub taxa is presented in Tables S8 and S9. (F) Degree and interaction type of the top 10 hub nodes in wild and cultivated rice networks. “Intra-kingdom correlation” refers to BB or FF, and “interkingdom correlation” refers to BF.

Interkingdom network analysis also showed that wild rice networks had more nodes and edges than cultivated rice, and a higher proportion of negative edges and modularity (proportion of negative edges/modularity: 19.3%/0.51) than cultivated rice networks (proportion of negative edges/modularity: 17.7%/0.47; Fig. 5A and Table S7). Meanwhile, wild rice networks exhibited a substantially higher average degree and clustering coefficient than cultivated rice, suggesting that the species in the former were more correlated and had more cooccurrence than the latter species (*P < *0.01) (Fig. 5B and C). Positive correlations dominated both the intrakingdom network (94.6% bacterial–bacterial [BB] and 71.7% fungal–fungal [FF] in the wild rice network, and 89.7% BB and 79% FF in the cultivated rice network) and the interkingdom correlations (72.7% and 68% of the bacterial–fungal [BF] network of wild rice and cultivated rice, respectively) (Fig. 5D). Further, the top hub node with the highest degree and closeness centrality values in the wild rice intrakingdom network was BASV180 (wild-enriched taxa, *Sphingomonas*), and that for cultivated rice was BASV51 (core taxa, Pseudomonas) (Fig. 5E, Tables S8 and S9). The edges of the top 10 hub nodes in the interkingdom networks were primarily positive with the other nodes, particularly in the wild rice network (Fig. 5E and F).

### The assembly processes of rice phyllosphere microbial communities.

**FIG 6:**
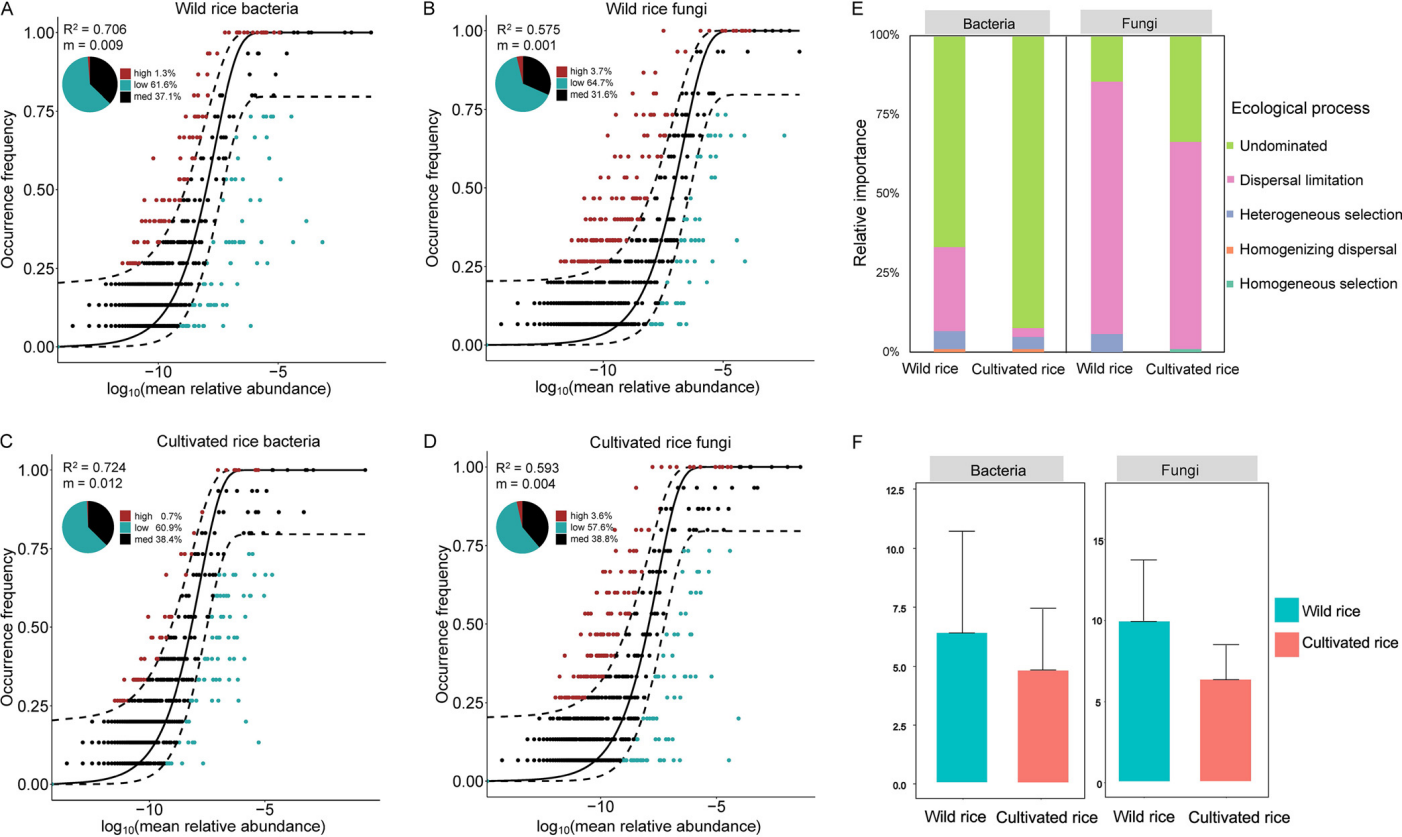
Community assembly of wild and cultivated rice phyllosphere microbial communities. Sloan neutral model prediction of wild rice bacterial (A) and fungal (B) communities and cultivated rice bacterial (C) and fungal (D) communities, indicated by R^2^ values (fit to neutral assembly process) and m values (estimated migration rate). ASVs are represented by data points and colored according to whether the taxon fit above (red), within (black), or below (blue) the 95% confidence interval (dashed lines). (E) The relative importance of five ecological processes of wild and cultivated rice phyllosphere microbial communities. (F) Comparison of mean community-level habitat niche breadths of bacterial and fungal taxa among wild and cultivated rice phyllosphere.

Neutral model analysis showed that a majority of the distribution of ASVs in the rice phyllosphere deviated from the neutral expectation (Fig. 6), which suggests that the contribution of deterministic processes to rice phyllosphere microbial communities was higher than that of stochastic processes. For bacteria, more wild rice taxa (62.9%) were identified to deviate from the neutral expectation than those of cultivated rice (61.6%) (Fig. 6A and C). Meanwhile, the degree of fit and migration rates (m values) of cultivated rice were higher than wild rice; all these results suggested that phyllosphere assembly of cultivated rice exhibited stronger migration and weaker dispersal limitation than that of wild rice (Fig. 6A and C). For fungi, the pattern is similar to bacteria, e.g., wild rice had more fungal taxa deviated from the neutral expectation, and lower m rates than cultivated rice (Fig. 6B and D). These results are consistent with the results of null models, which showed that the dispersal limitation and heterogeneous selection posed more influence on wild rice than on cultivated rice, and dispersal limitation was the most important process regulating the assembly of both wild and cultivated rice fungal communities (Fig. 6E). In addition, the wild rice phyllosphere exhibited higher bacterial and fungal community-level habitat niche breadths than cultivated rice, though the values were not statistically different (Fig. 6F).

## DISCUSSION

The core microbiome is the shared microbes among different rice cultivars and different geographical locations, which are considered to be coevolved with the host (8). Our results showed that many members of these core microbes have been reported to have the ability to enhance the plant growth. For example, *Pantoea*, the most abundant genus in both the wild and cultivated rice phyllosphere, have been reported to enhance the plant growth through promoting plant nitrogen fixation (29). The functional prediction results also confirmed that the rice core phyllosphere microbiome could be involved in nitrogen cycle processes (Fig. 4A). Some members of the core microbiomes can also enhance the plant growth through activating nutrient availability. For instance, some species of *Bacillus* and *Stenotrophomonas* can activate iron availability for plant uptake by producing siderophores (30), and Serratia sp. could increase Zn availability for wheat through solubilization of ZnO. Some members of the core microbiomes, like Pseudomonas and *Micrococcus*, can degrade complex organic pollutants to bio-fertilizers, which is consistent with the functional prediction of aromatic compound degradation by core BASVs (Fig. 4A) (31, 32). Notably, the organic degrading bacterium BASV51 (Pseudomonas) was not only the core taxon, but also the top hub taxon in the interkingdom network of the cultivated rice (Fig. 5F and Table S9). The overlap between the core taxa and network hubs further indicated that there are some microbial taxa recruited by the rice that may act as keystone taxa for rice growth. Additionally, some members of the core microbiomes can enhance the plant growth through enhancing plant resistance environmental stress. Some species of *Shewanella*, *Arthrobacter*, and *Microbacterium* are identified as highly salt stress-tolerant strains; *Curtobacterium* can improve the drought resistance and metal tolerance of plants (33, 34).

For the core fungi, many members (e.g., *Sporobolomyces*, *Cladosporium*, *Alternaria*, etc.) have also been frequently detected from the leaves and play diverse functions (Fig. 2 and Table S4) (35). For example, many strains of the *Papiliotrema* genus can protect different crops against fungal diseases (36), species of *Cladosporium* can enhance systemic stress tolerance for the host plant through producing active volatile organic compounds such as methyl benzoate (37), and some species of *Hasegawazyma* can enhance plant tolerance to toxic metals, such as Pb (38). Therefore, our study illustrates that the core phyllosphere microbiomes are diversely beneficial for rice growth, and further investigation on managing their community structure would improve sustainable rice production.

It is worth mentioning that most of the wild-enriched ASVs could be beneficial for plants to acquire nutrients, such as carbon cycling and nitrogen fixing (Fig. 4A and Table S5). For example, *Methylobacterium*, the most wild-enriched genus, can produce plant growth-promoting metabolites and promote plant nitrogen fixation (39). In addition, a recent study reported that host selection might drive specific *Methylobacterium*–rice associations, and phyllosphere *Methylobacterium* was demonstrated to be beneficial for rice yield under field conditions (40). Some wild-enriched microbial taxa are involved in stress resistance, such as soil contamination, drought, and UV radiation (Fig. 3 and Table S5). For example, salt stress-tolerant strains of *Shewanella*, *Arthrobacter*, and *Microbacterium*, drought-tolerant strains of *Curtobacterium*, and radiation-tolerant strains of *Kineococcus* are significantly enriched in wild rice compared with cultivated rice. Compared to the human-managed growth circumstances of cultivated rice, the wild rice phyllosphere microbiome needs to adapt to a much more hostile environment (41). Thus, these differences indicate that wild rice may have the ability to recruit beneficial bacteria for mutualistic symbiosis under the adversity.

Furthermore, compared with the fungal communities in cultivated rice, *Phaeosphaeria*, *Khuskia*, *Hannaella*, and *Setophoma* were highly enriched in the phyllosphere of wild rice, which also showed significantly higher functional potentials in promoting plant growth and against fungal parasites and animal pathogens (Fig. 3, 4B; Table S6). *Phaeosphaeria*, the most abundant taxa of wild-enriched FASVs, has shown antagonistic activities against plant pathogens by competing for nutrients or producing a wide array of secondary metabolites, including antimicrobials (42)*. Khuskia* is a saprophytic fungus, which can decompose plant cell walls and produce ligninases, xylanases, and/or Mn-peroxidase, helping plants to acquire nutrients (42). Members of the genus *Hannaella*, which are frequently observed in the phyllosphere of various plant species, are known to promote plant growth by producing indole acetic acid and decomposing organic matter (43). Notably, the potential fungal plant pathogens of cultivated rice were more than that of wild rice (Fig. 4B and Fig. S6); for example, Fusarium was significantly enriched in cultivated rice (Fig. 3, Table S6). The members of Fusarium can produce mycotoxin and cause root rot disease, and have been reported to be enriched in roots of modern cultivars of sunflower compared to nondomesticated sunflowers (44), suggesting that the domesticated crop may have lost the ability to prevent certain soilborne pathogens. Through comparing the abundance of microbes between the phyllospheres of wild and cultivated rice, and considering the possible effects of different soil chemical properties, irrigation/hydration levels, and even pesticide application on the differences in phyllosphere microbial communities, some beneficial phyllosphere microbes may have been lost during rice domestication. Moreover, several wild rice-enriched taxa, such as *Sphingomonas* and *Khuskia*, were also identified as hub taxa in the wild rice interkingdom network, which may be the keystone taxa and deployed to organize favorable plant microbiomes (Fig. 5F and Table S8) (45). Further investigation on recovering these lost taxa would be promising strategies for promoting rice production, and this study provides a list of the candidates for future researching.

Compared to other habitats such as the rhizosphere, the plant phyllosphere lacks water and nutrients and also suffers from large fluctuations in humidity and high levels of solar radiation (46). Thus, host genotype can act as an important plant-imposed filter by preventing the colonization of species that lack the abilities required to survive in the phyllosphere (35). Moreover, anthropogenic activities such as fertilization and pest control may also affect microbial assembly processes and might increase the dominance of stochastic processes. Previous studies have shown that the assembly of microbial communities in agricultural soils was more controlled by neutral processes than natural soils, indicating that long-term cultivation and management practices might enhance the stochastic influx and dispersal of microorganisms (41). This phenomenon is consistent with our results that the stochastic processes explained more variation of the cultivated rice phyllosphere community than that of wild rice (Fig. 6). In addition, our results showed that the dispersal limitation was the dominant process for the phyllosphere fungal community, and the migration rates of fungi were lower than bacteria in both wild and cultivated rice (Fig. 6). This difference between fungi and bacteria could be explained by their body size and lifestyle, and our results provide new evidence for the “size-dispersal” hypothesis (Fig. 6) (47).

Cooccurrence patterns have widely been used to explore the complex interaction webs and ecological rules for microbial community assembly within a specific ecological niche (45, 48, 49). Our study revealed that the correlations in all the microbial networks were predominantly positive (Fig. 5 and Fig. S5). These positive correlations indicate that there should be extensive cooperation, such as syntrophic or trophic relationships, between most microbial members that may share niches based on nutritional preferences and functional distinctiveness in the phyllosphere environment (35). Although positive correlations are predominant, fungal networks have higher proportions of negative relationships than bacterial networks (Fig. S5). It has been reported that fungal propagules may antagonize the growth of other phyllosphere members by producing metabolites, resulting in negative correlations (35). In addition, we found that the wild rice interkingdom network has more negative interactions than that of the cultivated rice (Fig. 5A), which indicates ecological competition and can increase the stability of microbial communities by reducing the destabilizing effects of cooperation (50). Moreover, the networks of wild rice have more interactions and higher connectivity and modularity than those of cultivated rice (Table S7). These results suggest that wild rice has a better-structured and more stable community than cultivated rice, which is possibly due to less anthropogenic interference or host genotypic difference (51). Additionally, significantly higher beneficial functional profiles were found in wild rice phyllosphere bacteria than in cultivated rice, most of which were involved in the nitrogen and carbon cycles (Fig. S3). This result is supported by previous studies that have also shown that more complex microbial coexistence patterns are closely related to higher functional profiles of the community (52). Therefore, to optimize the utilization of wild rice phyllosphere microbiota for sustainable agriculture in the future, we need further focus on not only the functions of individual microorganisms, but also the assembly processes and network relationships of the communities.

### Conclusions.

In summary, our findings demonstrate that the wild rice phyllosphere microbiome showed a significantly different preference compared to cultivated rice, which possesses more complicated and stable communities than cultivated rice. Deterministic assembly (heterogeneous selection) governed the wild rice phyllosphere microbiome more than the cultivated rice. Moreover, our study provides not only a list of core and potential beneficial microbes, but also several functional network profiles of wild rice. This information would be useful for the future utilization of rice phyllosphere microbiota to enhance crop performance and sustainability, especially in the framework of sustainable agroecosystems.

## MATERIALS AND METHODS

### Sampling.

Rice samples were collected from three regions in Wenchang (19° 47′ 12″ N, 110° 40′ 48″ E), Qionghai (19° 6′ 37″ N, 110° 28′ 55″ E), and Wanning (18° 44′ 25″ N, 110° 24′ 37″ E), in Hainan province, south China (Fig. S1). The three regions represent the main typical distribution area of wild rice in Hainan, which exhibits abundant genetic diversity of wild rice in China (53). Wild rice populations were sampled in natural reserves, and cultivated rice was sampled in the nearest paddy field at distances of  <1,000 m from the wild rice sampling site. In each region, five pairs of wild and cultivated rice were sampled during the flowering stage, indicating five replicates, and each replicate consisted of a composite sample by mixing leaves from five rice individuals. From each plant, 3 to 4 leaves, selected from the mid-upper part of the plant, were cut from near the stem using ethanol-sterilized scissors and stored in sterilized plastic bags (54). Then, the samples were immediately transported to the laboratory on dry ice and stored at −80°C until further experiment.

### DNA extraction.

The microbial DNA from the rice phyllosphere was extracted as previously described but with the following modifications (55). Around 3 g of rice leaf tissue (cut into pieces with sterile scissors) from each sample was weighed into a 250-mL conical flask containing 100 mL of 0.01 M sterile phosphate-buffered saline (pH = 7.4) and then mixed. The conical flasks were sonicated (frequency 40 kHz, power 150 W) in an ultrasonic cleaning bath (KQ-3200DE, Ultrasonic Instruments Co., Ltd., Kunshan) for 5 min, followed by shaking at 180 rpm at 30°C for 2 h. The obtained mixture was initially filtered through a sterilized nylon gauze followed by filtration through a 0.22-μM cellulose membrane, which was then cut into pieces and subjected to DNA extraction using a FastDNA Spin kit for soil (MP Biomedical, Santa Ana, USA) according to the manufacturer’s guidelines. The quality of the extracted DNA was assessed by electrophoresis in 1.5% agarose gel and with an ND-1000 spectrophotometer (NanoDrop Technology, Wilmington, USA). The DNA concentration was determined using a Qubit dsDNA HS assay kit (Thermo Fisher Scientific Inc, Waltham, USA). Finally, the DNA extracts of all samples were stored at −20°C prior to analysis.

### Amplicon sequencing and bioinformatic analysis.

The primer sets 515F/806R and ITS1F/ITS2R were used to amplify the bacterial 16S rRNA gene and fungal ITS region, respectively (56). PCR conditions were 94°C for 5 min to initial enzyme activation, followed by 35 amplification cycles of 94°C for 45 s, 50°C for 60 s, and 72°C for 90 s with a final elongation at 72°C for 10 min. The PCR mixtures contain 5× TransStart FastPfu buffer 4 μL, 2.5 mM dNTPs 2 μL, forward primer (5 μM) 0.8 μL, reverse primer (5 μM) 0.8 μL, TransStart FastPfu DNA polymerase 0.4 μL, template DNA 10 ng, and finally ddH_2_O up to 20 μL. PCR products were purified, pooled, and sequenced on the Illumina Miseq 2500 platform (Majorbio, Shanghai, China). The raw sequences were discarded if they contained ambiguous nucleotides, with a low (Q <20) quality score, and short in length (<100 bp). The table generation of amplicon sequence variants (ASVs) was conducted following the DADA2 pipeline in R 4.0 (57) and the package dada2 (v1.12.1). The ASV sequences that showed poor alignment performance were discarded. Taxonomic identification of bacteria and fungi was obtained against the SILVA (v138) (58) and UNITE (v8.0) databases (59), respectively. Bacterial sequences that match host mitochondria and chloroplast were removed.

### Statistical analysis.

All statistical analyses were performed using R software. Significant differences between groups were tested by analysis of variance (ANOVA) or Wilcoxon with false discovery rate (FDR) adjusted *P* value. Principal coordinate analysis (PCoA) and permutational multivariate analyses of variance (PERMANOVA) based on the Bray-Curtis distance were performed to assess the differences in community composition using the “labdsv” and “vegan” packages (60, 61). The core microbial taxa were primarily selected from the ASVs appearing (100% of prevalence, relative abundance >0.1%) among the two rice varieties across all sites. Heatmap plotting and Venn diagrams were performed in R 3.2.3 with the “pheatmap” package and “VennDiagram” packages. Differences in the abundance were considered significant when FDR-adjusted *P* values were lower than 0.01. Differentially abundant bacterial ASVs (BASVs) and fungal ASVs (FASVs) were identified by the DESeq2 package and visualized in Volcano plots with Excel 2016. Sequences were aligned with MUSCLE using default parameters, and MEGA-X was used to construct a neighbor-joining tree (62, 63). The bootstrap consensus tree was inferred from 1,000 replicates and visualized by ITOL (64). Bacterial functional profiles were predicted using functional annotation of prokaryotic taxa (FAPROTAX) (65). Fungal pathogens and functional guilds were inferred (guild assignments with confidence rankings “Highly probable” and “Probable” were retained) using the program FUNGuild (66).

The assembly processes of bacterial and fungal communities were investigated by both neutral and null models. First, to determine the potential importance of stochastic processes on community assembly, the Sloan neutral community model was used to predict the relationship between ASV detection frequency and their relative abundance (67). Second, a null-modeling-based statistical framework was used to quantify the contributions of various ecological processes to bacterial community structure (27). In addition, Levins’ niche breadth index was conducted using the *niche.width* function in the *spaa* package in R.

Since the number of individual ASVs varied significantly across different samples, we used the relative abundances of the ASVs to construct a molecular ecological network (68). Before network inference, rare ASVs with relative abundance of less than 0.01% were removed to mitigate zero inflation. Network analysis was performed using R with the Vegan and psych packages. The correlation matrix was constructed by calculating all possible pairwise Spearman’s rank correlations of microbial taxa. We considered a cooccurrence pattern between microbial taxa to be robust if the absolute correlation coefficient was greater than 0.6 with a *P* value <0.01. The *P* values were adjusted with a multiple testing correction using the Benjamini-Hochberg method to reduce the chances of obtaining false-positive results. To improve the representativeness of the ASVs and reduce the network complexity, only significant pairwise correlations were used to construct the network, with each node representing an ASV and each edge representing a significant association between the ASVs. Networks were visualized using the Gephi platform (v0.9.1). Based on theoretical modeling and simulation data, network stability was measured by the proportion of negative or positive correlations and the modularity. Microbial networks having the properties of greater modularity, lower positive correlations among members, and higher negative correlations among members are considered to be more stable (50, 69, 70).

### Data availability.

The original sequences have been deposited in the NCBI Sequence Read Archive (SRA) under BioProject ID number PRJNA888831.
