# Supplementary material for: Distinctive Structure and Assembly of Phyllosphere Microbial Communities between Wild and Cultivated Rice
Source: Microbiol Spectr. 2023 Jan 10;11(1):e04371-22. doi: 10.1128/spectrum.04371-22 (PMC9927517; doi:10.1128/spectrum.04371-22)
Supplement: Supplemental file 1 — Supplemental material. Download spectrum.04371-22-s0001.pdf, PDF file, 0.4 MB [file spectrum.04371-22-s0001.pdf]

Supporting Information for

Distinctive structure and assembly of phyllosphere microbial communities between wild and cultivated rice

Yue Yin[1,2], Yi-Fei Wang[1,2], Hui-Ling Cui[1,2], Rui Zhou[1,2], Lv Li[1,2], Gui-Lan Duan[1,2*], Yong-Guan Zhu[1,2,3]

1. State Key Lab of Urban and Regional Ecology, Research Center for Eco-Environmental Sciences, Chinese Academy of Sciences, Beijing 100085, China
2. University of Chinese Academy of Sciences, Beijing 100049, China
3. Institute of Urban Environment, Chinese Academy of Sciences, Xiamen 361021, China

----

*Corresponding author. E-mail: duangl@rcees.ac.cn (Gui-Lan Duan).

Table S1. Relative importance of site and rice cultivar on phyllosphere bacterial community composition revealed through PERMANOVA.

Variable	Degrees of freedom	Sum of squares	*F*	R^2	*P*
Site	2	1.214	4.443	0.209	0.001
Rice cultivar	1	0.775	5.670	0.133	0.001
Site * Rice cultivar	2	0.547	2.001	0.094	0.022
Residuals	24	3.278		0.564	
Total	29	5.813		1.000	

Table S2. Relative importance of site and rice cultivar on phyllosphere fungal community composition revealed through PERMANOVA.

Variable	Degrees of freedom	Sum of squares	*F*	R^2	*P*
Site	2	3.245	13.564	0.361	0.001
Rice cultivar	1	1.166	9.745	0.130	0.001
Site * Rice cultivar	2	1.696	7.090	0.189	0.001
Residuals	24	2.871		0.320	
Total	29	8.977		1.000	

Table S3. The relative abundance of shared core bacterial genus of the wild rice and cultivated rice phyllosphere. Asterisks represent statistical significances between wild and cultivated rice as determined by FDR-corrected Wilcoxon test (*p < 0.05; **p < 0.01).

Shared core bacterial genus	Relative abundance in wild rice	Relative abundance in cultivated rice	Relative abundance in all samples	Corresponding phylum
Pantoea*	0.3297	0.5251	0.4274	Proteobacteria
Curtobacterium*	0.1437	0.0551	0.0994	Actinobacteriota
Exiguobacterium	0.1092	0.0393	0.0743	Firmicutes
Methylobacterium**	0.0590	0.0367	0.0479	Proteobacteria
Pseudomonas	0.0129	0.0593	0.0361	Proteobacteria
Acinetobacter	0.0540	0.0047	0.0293	Proteobacteria
Serratia	0.0427	0.0001	0.0214	Proteobacteria
Klebsiella	0.0318	0.0034	0.0176	Proteobacteria
Sphingomonas	0.0210	0.009	0.015	Proteobacteria
Xanthomonas	0.0219	0.0052	0.0135	Proteobacteria
Comamonas**	0.0013	0.018	0.0097	Proteobacteria
Solibacillus	0.0007	0.0122	0.0065	Firmicutes
Shewanella*	0.0004	0.0124	0.0064	Proteobacteria
Arthrobacter	0.0061	0.0065	0.0063	Actinobacteriota
Hymenobacter	0.0097	0.0002	0.0049	Bacteroidota

Stenotrophomonas*	0.0002	0.0092	0.0047	Proteobacteria
Micrococcus	0.0030	0.0051	0.004	Actinobacteriota
Microbacterium	0.0034	0.0046	0.004	Actinobacteriota
Gemmobacter	0.0006	0.0072	0.004	Proteobacteria
Bacillus	0.0029	0.0047	0.0038	Firmicutes
Kineococcus**	0.0057	0.0018	0.0037	Actinobacteriota
Pseudochrobactrum	0.0001	0.0051	0.0026	Proteobacteria

Table S4. The relative abundance of shared core fungal genus of the wild rice and cultivated rice phyllosphere. Asterisks represent statistical significances between wild and cultivated rice as determined by FDR-corrected Wilcoxon test (*p < 0.05; **p < 0.01).

Shared core fungal genus	Relative abundance in wild rice	Relative abundance in cultivated rice	Relative abundance in all samples	Corresponding class
Nigrospora**	0.0152	0.2492	0.1322	Sordariomycetes
Pyrenophora	0.1112	0.1499	0.1305	Dothideomycetes
Papiliotrema	0.1688	0.0803	0.1246	Tremellomycetes
Phaeosphaeria**	0.0777	0.012	0.0448	Dothideomycetes
Sporobolomyces	0.022	0.0574	0.0397	Microbotryomycetes
Neoascochyta	0.0751	0.0025	0.0388	Dothideomycetes
Symmetrospora	0.0447	0.0321	0.0384	Cystobasidiomycetes
Cladosporium	0.0217	0.04	0.0308	Dothideomycetes
Pyrenochaetopsis	0.016	0.0306	0.0233	Dothideomycetes
Hannaella	0.0147	0.0294	0.0221	Tremellomycetes
Saitozyma	0.0187	0.0188	0.0188	Tremellomycetes
Curvularia	0.0097	0.022	0.0159	Dothideomycetes
Hasegawazyma	0.0203	0.0064	0.0134	Cystobasidiomycetes
Edenia	0.0026	0.0072	0.0049	Dothideomycetes
Alternaria	0.0012	0.0009	0.0011	Dothideomycetes

Table S5. The relative abundance and related information of main enriched phyllosphere bacterial ASVs of wild rice and cultivated rice, respectively (relative abundance > 0.1%).
[d]Name of family to which this incertae sedis genus belongs.

ASV ID	Enriched	log_2FoldChange	*P* value	Relative Abundance	Genus
BASV18	Wild Rice	-25.4939	0.001	0.021887	Xanthomonas
BASV22	Wild Rice	-23.5313	0.001	0.007475	Enterobacter
BASV130	Wild Rice	-22.99	0.001	0.001338	Sphingomonas
BASV75	Wild Rice	-21.2825	0.001	0.004292	Hymenobacter

BASV354	Wild Rice	7.119971	0.003	0.000187	Methylocystaceae[d]
BASV68	Wild Rice	-6.93062	0.003	0.004646	Beijerinckia
BASV106	Wild Rice	-5.00733	0.012	0.002148	Amnibacterium
BASV43	Wild Rice	-4.72411	0.002	0.009682	Hymenobacter
BASV15	Wild Rice	-4.70803	0.001	0.024008	Methylobacterium
BASV80	Wild Rice	-4.13386	0.003	0.003609	Intrasporangiaceae[d]
BASV81	Wild Rice	-3.63691	0.005	0.002813	Sphingomonas
BASV20	Wild Rice	-3.05116	0.001	0.015292	Methylobacterium
BASV45	Wild Rice	-2.75036	0.008	0.007132	Methylobacterium
BASV31	Cultivated Rice	3.711015	0.001	0.007244	Gemmobacter
BASV14	Cultivated Rice	3.7624	0.001	0.01804	Comamonadaceae[d]
BASV74	Cultivated Rice	3.778559	0.003	0.003884	Brevundimonas
BASV36	Cultivated Rice	3.8028	0.001	0.009273	Acinetobacter
BASV78	Cultivated Rice	4.478526	0.004	0.003756	Stenotrophomonas
BASV119	Cultivated Rice	4.735162	0.046	0.001403	Brevundimonas
BASV73	Cultivated Rice	5.156821	0.005	0.003961	Aeromonas
BASV52	Cultivated Rice	5.16912	0.014	0.006921	Pseudomonas
BASV85	Cultivated Rice	5.19378	0.002	0.003397	Pseudomonas
BASV44	Cultivated Rice	5.24227	0.003	0.009233	Stenotrophomonas
BASV65	Cultivated Rice	5.27019	0.001	0.004488	Pseudomonas
BASV32	Cultivated Rice	5.345035	0.002	0.012376	Shewanella
BASV60	Cultivated Rice	5.366723	0.001	0.005054	Pseudochrobactrum
BASV94	Cultivated Rice	5.819216	0.02	0.002895	Pseudomonas
BASV116	Cultivated Rice	5.93813	0.01	0.001268	Comamonas
BASV86	Cultivated Rice	6.072576	0.008	0.003537	Pseudomonas
BASV125	Cultivated Rice	6.153919	0.013	0.001383	Pseudomonas
BASV82	Cultivated	6.964656	0.003	0.003654	Stenotrophomonas

	Rice				
BASV58	Cultivated Rice	25.87722	0.001	0.005157	Xanthomonas

Table S6. The relative abundance and related information of main enriched phyllosphere fungal ASVs of wild rice and cultivated rice, respectively (relative abundance > 0.1%).
[d]Name of family to which this incertae sedis genus belongs.

ASV ID	Enriched	$\log_2$FoldChange	*P* value	Relative Abundance	Genus
FASV53	Wild rice	-27.9984	0.001	0.011949	Khuskia
FASV144	Wild rice	-26.7133	0.001	0.002316	Acidomyces
FASV193	Wild rice	-26.0028	0.001	0.001352	Pyrenophora
FASV135	Wild rice	-24.8089	0.001	0.002621	Microdochium
FASV185	Wild rice	-24.2512	0.001	0.001504	Mycosphaerellaceae[d]
FASV96	Wild rice	-23.5736	0.001	0.004343	Khuskia
FASV155	Wild rice	-22.2582	0.001	0.001658	Papiliotrema
FASV68	Wild rice	-10.8758	0.001	0.002335	Khuskia
FASV86	Wild rice	-10.4141	0.001	0.004996	Khuskia
FASV35	Wild rice	-9.30141	0.001	0.00104	Khuskia
FASV121	Wild rice	-9.22545	0.001	0.002913	Phaeosphaeriaceae[d]
FASV64	Wild rice	-8.97258	0.001	0.007482	Setophoma
FASV134	Wild rice	-6.97087	0.001	0.002574	Setophoma
FASV167	Wild rice	-6.92026	0.001	0.00174	Echria
FASV65	Wild rice	-6.63776	0.001	0.005361	Phialemonium
FASV40	Wild rice	-6.43404	0.001	0.016362	Hannaella
FASV73	Wild rice	-6.30849	0.001	0.006517	Setophoma
FASV177	Wild rice	-6.11818	0.001	0.001343	Ascochyta
FASV168	Wild rice	-6.04242	0.001	0.001704	Phaeosphaeriaceae[d]
FASV183	Wild rice	-5.78717	0.001	0.001414	Mycosphaerellaceae[d]
FASV98	Wild rice	-5.77346	0.001	0.004172	Curvularia
FASV131	Wild rice	-5.75957	0.001	0.002621	Phaeosphaeria
FASV13	Wild rice	-5.2933	0.001	0.007514	Leptosphaerulina
FASV105	Wild rice	-4.85856	0.001	0.003778	Phaeosphaeriaceae[d]
FASV178	Wild rice	-4.33513	0.001	0.001492	Mycosphaerellaceae[d]
FASV18	Wild rice	-4.03552	0.001	0.052973	Phaeosphaeria
FASV145	Wild rice	-2.28668	0.001	0.001007	Dokmaia
FASV133	Cultivated rice	2.503936	0.001	0.002128	Curvularia
FASV45	Cultivated	3.496055	0.001	0.009183	Rhodotorula

	rice				
FASV9	Cultivated rice	3.893596	0.001	0.01131	Hannaella
FASV78	Cultivated rice	4.420724	0.001	0.00482	Didymosphaeriaceae[d]
FASV2	Cultivated rice	4.570342	0.001	0.249172	Chaetomium
FASV77	Cultivated rice	5.751525	0.001	0.003142	Fusarium
FASV214	Cultivated rice	6.245437	0.001	0.001093	Dipodascus
FASV8	Cultivated rice	25.19561	0.001	0.00141	Hannaella

Table S7. The topological features of co-occurrence networks for different groups of microbial taxa in wild rice and cultivated rice phyllosphere.

Habitats	Microbial groups	Node[a]	Edge[b]	Average degree[c]	Average Clustering coefficient[d]	Average path length[e]	Diameter[f]	Modularity[g]	Graph density[h]
Wild rice	Bacteria	249	3402	27.325	0.584	2.948	10	0.343	0.11
	Fungi	293	4727	32.266	0.642	2.915	9	0.702	0.111
	Bacteria&fungi	380	8346	43.926	0.615	2.727	10	0.511	0.116
Cultivated rice	Bacteria	240	2974	24.783	0.578	2.832	7	0.531	0.104
	Fungi	254	2734	21.528	0.583	2.946	8	0.593	0.085
	Bacteria&fungi	331	5489	33.166	0.564	2.752	7	0.47	0.101

[a]Microbial taxon (at ASV level) with at least one significant ($P < 0.01$) and strong (Spearman's correlation coefficient > 0.6 or < −0.6) correlation.
[b]Number of connections/correlations obtained by Spearman analysis.
[c]Node connectivity; shows how many connections (on average) each node has to another unique node in the network.
[d]How nodes are embedded in their neighbourhood, and thus the degree to which they tend to cluster together.
[e]Average network distance between all pairs of nodes. It indicates the number of steps one needs to make on average in the graph in order to connect two randomly selected nodes.
[f]The longest distance between the nodes that exists in the network.
[g]The Capability of the nodes to form highly connected communities, that is, a structure with high density of between nodes connections.
[h]The intensity of connections among nodes

Table S8. Taxonomic information of the top 10 hub taxa in wild rice interkingdom network.

ASV ID	Degree	Classification	Taxonomy information
BASV180	130	wild-enriched	p__Proteobacteria; c__Alphaproteobacteria; o__Sphingomonadales; f__Sphingomonadaceae; g__Sphingomonas
BASV128	129	others	p__Proteobacteria; c__Gammaproteobacteria; o__Pseudomonadales; f__Moraxellaceae; g__Acinetobacter
BASV113	129	others	p__Proteobacteria; c__Gammaproteobacteria; o__Pseudomonadales; f__Moraxellaceae; g__Acinetobacter
FASV526	129	others	d__Fungi;p__Ascomycota;c__Dothideomycetes;o__Pleosporales;f__Tetraplosphaeriaceae;g__Tetraplosphaeria;s__Tetraplosphaeria_yakushimensis
BASV130	125	wild-enriched	p__Proteobacteria; c__Alphaproteobacteria; o__Sphingomonadales; f__Sphingomonadaceae; g__Sphingomonas
BASV148	125	others	p__Proteobacteria; c__Alphaproteobacteria; o__Rhizobiales; f__Beijerinckiaceae; g__1174-901-12; s__uncultured bacterium
FASV200	125	others	p__Basidiomycota;c__Tremellomycetes;o__Tremellales;f__Trimorphomycetaceae;g__Saitozyma;s__Saitozyma_flava
FASV531	124	others	p__Basidiomycota;c__Microbotryomycetes;o__Sporidiobolales;f__Sporidiobolaceae;g__Sporobolomyces;s__Sporobolomyces ruber
FASV111	124	others	p__Ascomycota;c__Dothideomycetes;o__Pleosporales;f__Phaeosphaeriaceae;g__unidentified;s__unidentified
FASV238	123	wild-enriched	p__Basidiomycota;c__Tremellomycetes;o__Tremellales;f__Trimorphomycetaceae;g__Khuskia;s__unidentified

Table S9. Taxonomic information of the top 10 hub taxa in cultivated rice interkingdom network.

ASV ID	Degree	Classification	Taxonomy information
BASV51	97	core taxa	p__Proteobacteria; c__Gammaproteobacteria; o__Pseudomonadales; f__Pseudomonadaceae; g__Pseudomonas
FASV93	97	others	p__Ascomycota;c__unclassified Ascomycota; o__unclassified Ascomycota; f__unclassified Ascomycota; g__unclassified Ascomycota; s__unclassified Ascomycota
BASV169	96	cultivated	p__Proteobacteria; c__Alphaproteobacteria; o__Rhodobacterales; f__Rhodobacteraceae; g__Gemmobacter
FASV25	95	others	p__Ascomycota;c__Dothideomycetes;o__Capnodiales;f__Mycosphaerellaceae; g__Septoria;s__Septoria_cretae
BASV176	95	cultivated	p__Bacteroidota; c__Bacteroidia; o__Bacteroidales; f__Tannerellaceae; g__Macellibacteroides
BASV152	95	others	p__Proteobacteria; c__Gammaproteobacteria; o__Xanthomonadales; f__Xanthomonadaceae; g__Stenotrophomonas
FASV383	94	others	p__Ascomycota;c__Sordariomycetes;o__Glomerellales;f__Plectosphaerellaceae;g__Plectosphaerella;s__unclassified Plectosphaerella
FASV510	94	others	p__Ascomycota;c__Dothideomycetes;o__Pleosporales;f__Pleosporaceae; g__Bipolaris; s__unclassified Bipolaris
FASV111	93	others	p__Ascomycota;c__Dothideomycetes;o__Pleosporales;f__Phaeosphaeriaceae; g__unidentified;s__unidentified
FASV309	93	others	p__Ascomycota;c__Dothideomycetes;o__Pleosporales;f__Morosphaeriaceae; g__Acrocalymma;s__unidentified

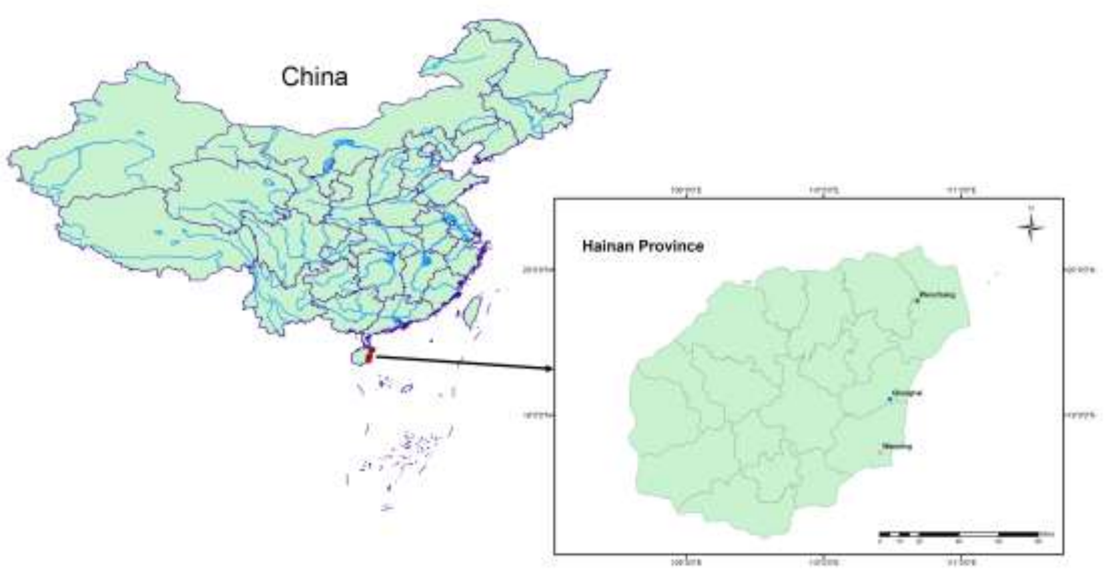

Figure S1. The location of sampling sites. Each circle represents a sampling site. The x and y axes represent longitude and latitude, respectively.

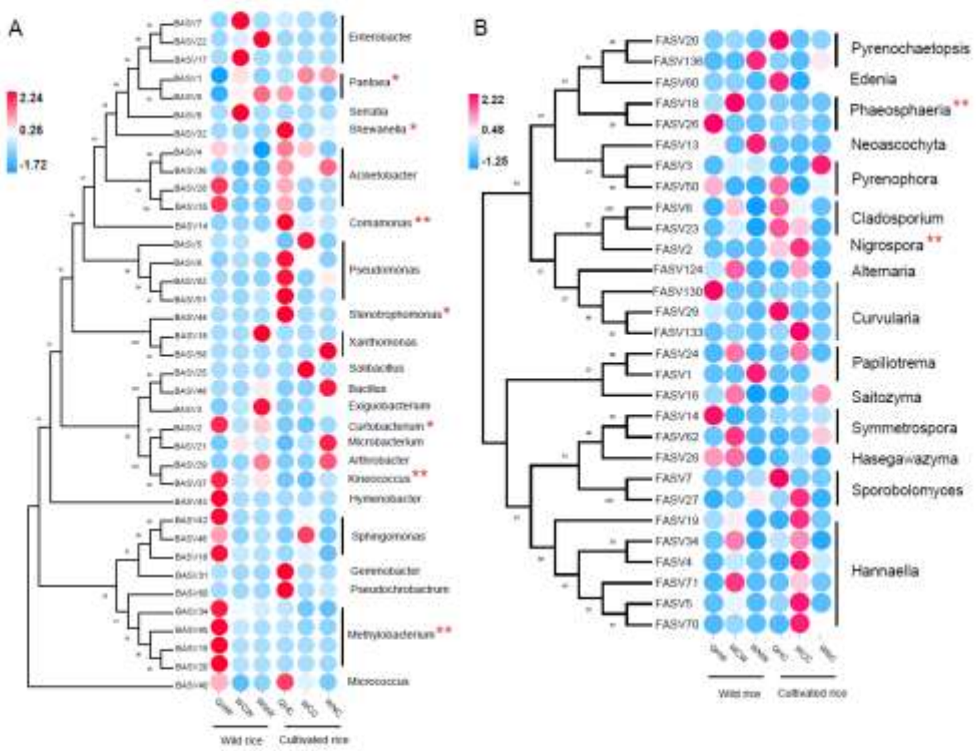

Figure S2. The shared core microbial taxa between wild and cultivated rice from different sites. Neighbor-joining (NJ) phylogenetic trees illustrating the 37 core bacterial (A) and 29 core fungal (B) ASVs (with 95% similarity as the cutoff value), respectively. The corresponding heat maps are coloured by log2-transformed fold changes and represent enrichments of specific bacterial and fungal ASVs in different groups. Asterisks represent statistical significances between wild and cultivated rice as determined by FDR-corrected Wilcoxon test (*$p < 0.05$; **$p < 0.01$). (Abbreviations: QHW, wild rice in Qionghai; WCW, wild rice in Wenchang; WNW, wild rice in Wanning; QHC, cultivated rice in Qionghai; WCC, cultivated rice in Wenchang; WNC, cultivated rice in Wanning).

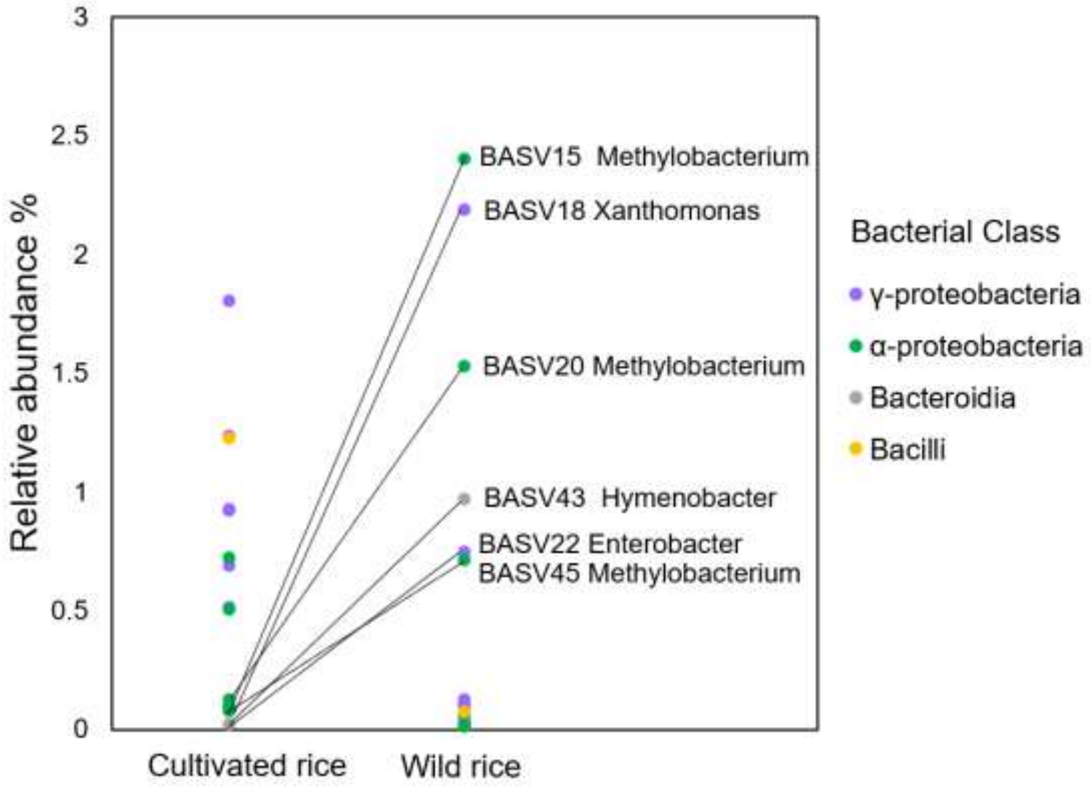

Figure S3. Several bacterial ASVs enriched in the wild rice were also identified as the core bacterial taxa in both wild and cultivated rice.

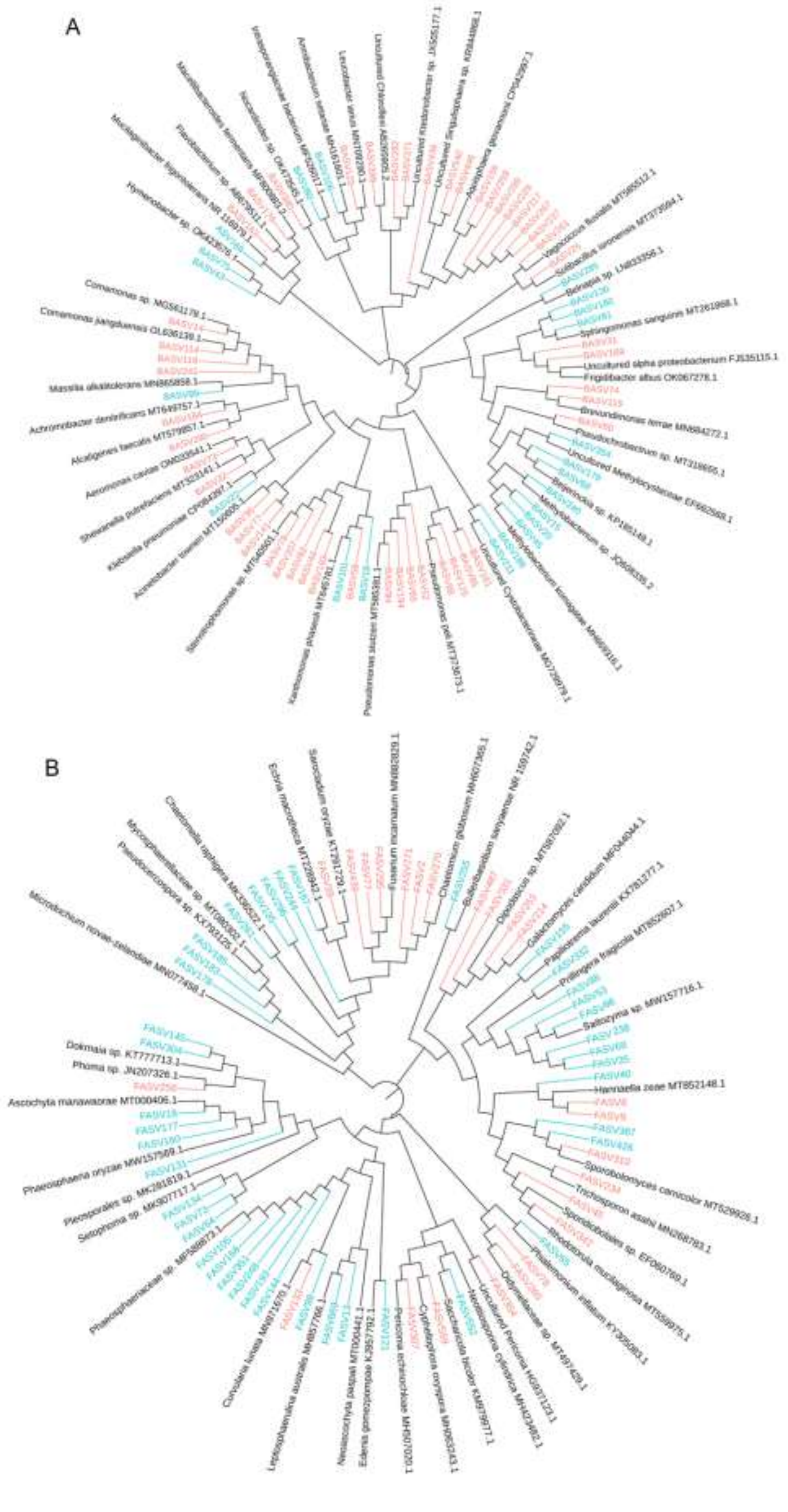
A
Uncultured alpha proteobacterium FJ535115.1
Frigidibacter albus OK067278.1
B
Hannaella zeae MT852148.1
Ascochyta manawaorae MT000406.1

Figure S4. Neighbor-joining (NJ) phylogenetic trees illustrating the 71 bacterial (A) and 65 fungal ASVs (B) which were significantly varied in wild and cultivated rice, respectively.

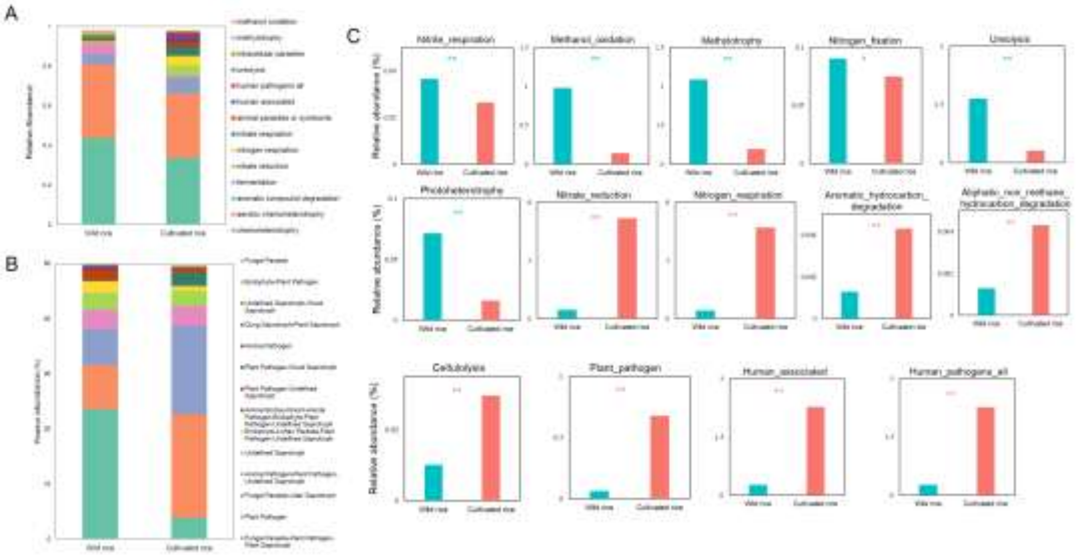

Figure S5. Predicted functional profiles of rice phyllosphere microbiome. (A) Functional prediction of bacterial communities in wild rice and cultivated rice using FAPROTAX. (B) Functional prediction of fungal communities in wild rice and cultivated rice using FUNGuild. (C) Functional prediction of bacterial communities in wild rice and cultivated rice using FAPROTAX. Blue asterisks indicate the significantly higher values in wild rice (*p < 0.05; **p < 0.01; FDR-corrected Wilcoxon rank-sum test); pink asterisks indicate significantly higher values in cultivated rice.

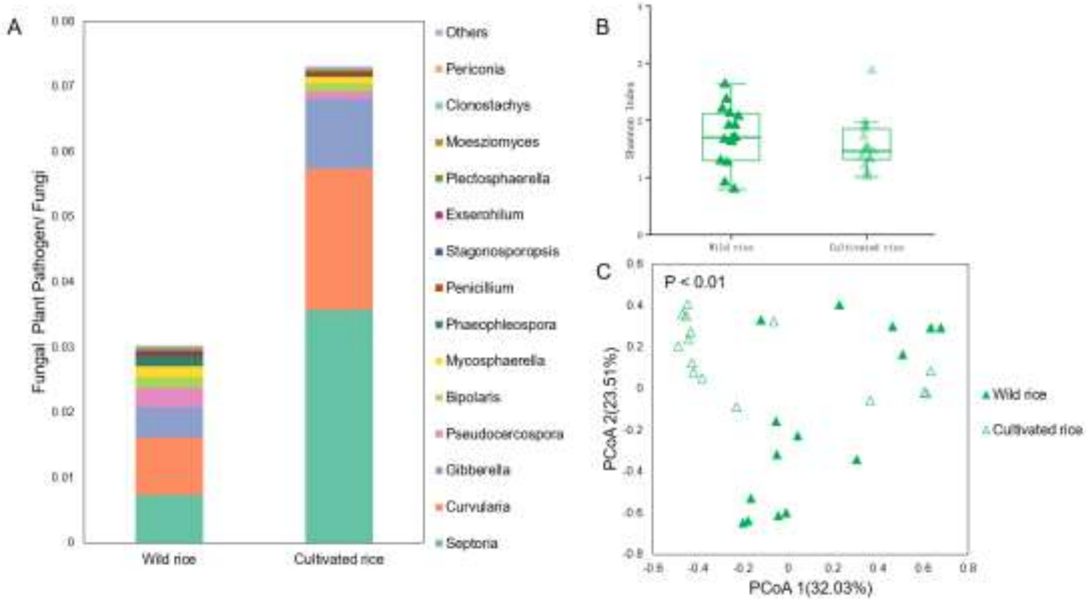

Figure S6. Taxonomic profiles of fungal plant pathogens in the rice phyllosphere. (A) the relative abundance of fungal plant pathogens in the wild and cultivated rice phyllosphere. (B) the Shannon index of fungal plant pathogens. (C) the PCoA analysis

of fungal plant pathogenic communities based on Bray−Curtis distances grouped by rice cultivars.

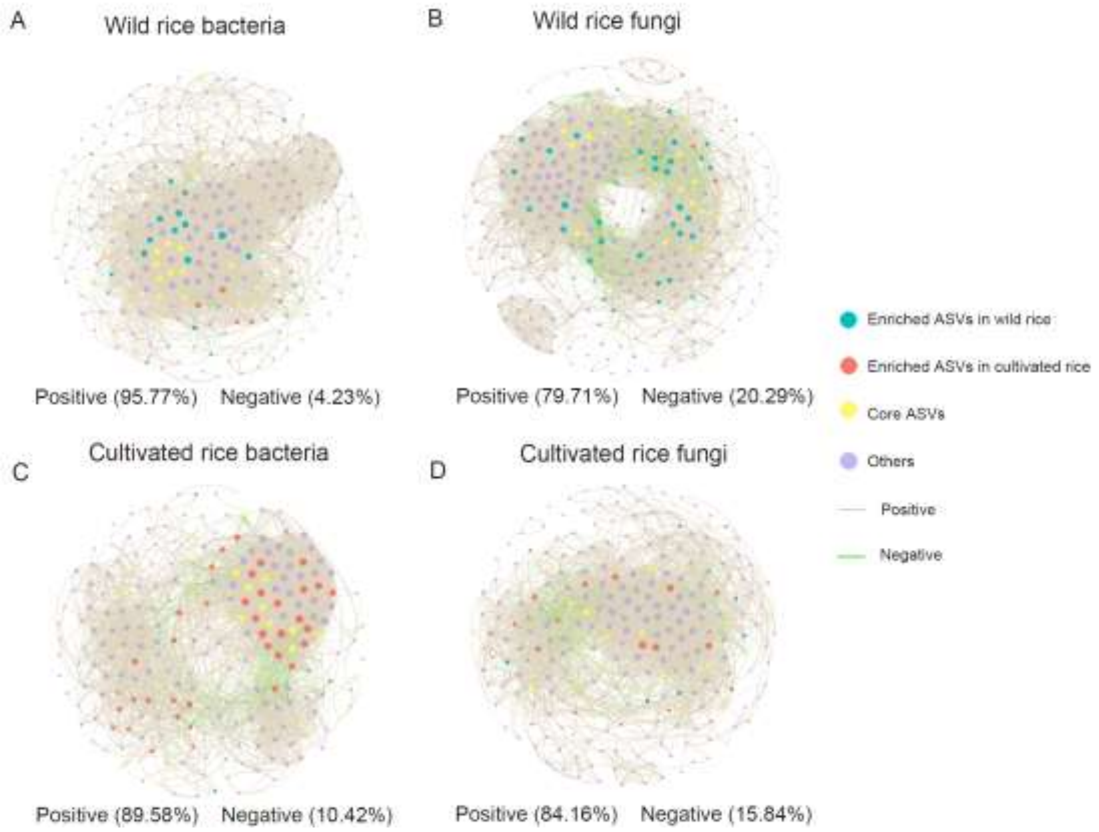

Figure S7. Network analysis revealing the co-occurrence patterns between microbiome (ASV level). A connection indicates a strong (Spearman's $\rho > 0.6$ or < -0.6) and significant (FDR-corrected $p < 0.01$) correlation. The size of each node is proportional to the degree of the ASVs. Positive and negative relationships are illustrated in gray and green, respectively. Blue, pink, yellow, and purple nodes represent wild-enriched, cultivated-enriched, shared core ASVs and others, respectively. (A) The co-occurrence patterns between wild rice bacteria. (B) The co-occurrence patterns between wild rice fungi. (C) The co-occurrence patterns between cultivated rice bacteria. (D) The co-occurrence patterns between cultivated rice fungi.
